# Associations between the thyroid panel and serum protein concentrations across pregnancy

**DOI:** 10.1038/s41598-021-94358-3

**Published:** 2021-08-05

**Authors:** Barbara Lisowska-Myjak, Agnieszka Strawa, Hanna Zborowska, Artur Jakimiuk, Ewa Skarżyńska

**Affiliations:** 1 Department of Biochemistry and Clinical Chemistry, Medical University of Warsaw, ul. Banacha 1, 02-097 Warsaw, Poland; 2 Department of Laboratory Diagnostics, Medical University of Warsaw, Warsaw, Poland; 3 Department of Reproductive Health, Institute of Mother and Child and Department of Obstetrics and Gynecology, Central Clinical Hospital of the Ministry of the Interior and Administration, Warsaw, Poland

**Keywords:** Physiology, Endocrinology

## Abstract

Establishing any characteristic associations between the serum parameters of thyroid function and serum proteins in pregnancy may aid in elucidating the role of the thyroid gland in the regulation of pregnancy-specific metabolic processes and in selecting candidate biomarkers for use in their clinical assessment. Concentrations of thyroid stimulating hormone (TSH), free tri-iodothyronine (fT3) and free thyroxine (fT4), six electrophoretically separated protein fractions (albumin, alpha-1-, alpha2-, beta-1-, beta-2- and gamma-globulins), representative proteins—albumin (ALB), transferrin (TRF), alpha-2-macroglobulin (AMG) and ceruloplasmin (CER) were measured in 136 serum samples from 65 women in their consecutive trimesters of pregnancy. The concentrations of TSH, fT4 and fT3 were significantly correlated (p < 0.05) with the concentrations of the albumin, alpha-2- and beta-1 globulin fractions. Significant correlations (p < 0.05) which were positive between fT4 and ALB and negative between fT4 and TRF were established throughout pregnancy. Significant negative correlations (p < 0.05) were demonstrated for fT3 with alpha-2-globulin, AMG and CER. Changes in the serum concentrations of thyroid hormones seen between the trimesters were found to correlate with the concentrations of high-abundance serum proteins. Opposite directions of correlations between fT4 and ALB and fT4 and TRF observed throughout pregnancy may indicate the shared biological role of these parameters in maintaining maternal homeostasis and they suggest their potential use in the clinic as a simple biomarker panel. A negative correlation of fT3 with CER in the second trimester possibly reflects their involvement in the active regulation of metabolic processes.

## Introduction

Thyroid hormones can potentially stimulate metabolic processes which involve numerous proteins detected in the blood serum^1–3^. Endocrine changes in pregnancy are reflected in the differences in the reference ranges for the thyroid panel (TSH, fT4, fT3, fT3/fT4) between pregnant and non-pregnant women^4–6^. Pregnancy also induces changes in anabolic and catabolic processes which may be accompanied by a characteristic profile of the qualitative and quantitative composition of maternal serum proteins^6^. Estrogen-dependent changes in serum levels of individual proteins have been demonstrated during pregnancy, including increases in carrier proteins for thyroid hormones^7^. A proteomic analyses performed so far demonstrated the effect of thyroid hormones on the regulation of selected plasma proteins^8^.

Identification of the effects of changes in the serum concentrations of thyroid hormones on the of the qualitative and quantitative composition of serum proteins in pregnancy could lead to better understanding of the role of the thyroid gland in the management of metabolic pathways to maintain homeostasis. When confirmed, these putative associations between the thyroid panel and changes in the serum concentrations of individual proteins with known biological properties and roles may provide more detailed information and clarify the physiological and pathological mechanisms which regulate the shared involvement of thyroid hormones and serum proteins in pregnancy-associated metabolic processes specific to each trimester.

Electrophoresis has proved to be a useful screening method to separate high-abundance proteins whose concentrations in the serum of pregnant women have effect on the quantification of electrophoretic fractions^9^. Variations in the concentration of proteins in the electrophoretic fractions are dependent on just 22 proteins which make up to 99% of the blood proteome^10^. Each electrophoretically separated fraction is composed of proteins at different concentrations, which are independently involved in different metabolic processes. Identification of individual proteins in these fractions, whose concentrations appear to be associated with the parameters of thyroid function may be an alternative method to characterize normal and abnormal pregnancyassociated metabolic processes which involve thyroid hormones and a source of novel biomarkers for their evaluation.

The aim of the study was to determine whether there were any associations between the variations in the concentrations of proteins contained in the electrophoretic fractions and the levels of thyroid function parameters (TSH, fT4, fT3, fT3/fT4 ratio) and to identify and measure the concentrations of individual proteins in these fractions as candidate biomarkers of thyroid function in pregnancy.

## Material and methods

### Subjects

Prospectively screened 65 healthy women aged 17–43 years (mean age ± SD: 31.4 ± 5.8) attending three routine antenatal visits in each trimester of normal singleton pregnancy: first trimester, pregnancy weeks 8–12 (n = 55); second trimester, pregnancy weeks 20–24 (n = 42); third trimester, pregnancy weeks 34–38 ( n = 39). The same women were assessed in each trimester. The differences in the concentrations of parameters in the serum were analyzed between the groups representing subsequent stages of pregnancy of the same women. Gestational age was calculated from the first day of the last menstrual period and confirmed by clinical examination. Clinical, laboratory and ultrasound examinations were used to confirm a normal pregnancy. The pregnant women were non-smokers and did not receive any anti-inflammatory medication. Infections and any other health problems were the exclusion criteria for participation in the study.

### Blood samples

Blood samples were drawn by venipuncture into test tubes which did not contain an anticoagulant and allowed to clot at room temperature. After centrifugation at 3000 g for 10 min at 4º C, serum was obtained and aliquots were immediately stored at – 80ºC until assayed. On the day of the measurements serum samples were thawed at room temperature using gentle vortexing.

### Methods

*Serum concentrations of the thyroid hormones* were measured by the chemiluminescence method using the COBAS 800 analyzer (Roche Diagnostics, Basel, Switzerland) and the dedicated reagents.

*The serum protein components were separated by electrophoresis into six fractions* (albumin, alpha-1 globulins, alpha-2 globulins, beta-1 globulins, beta-2 globulins and gammaglobulins) using the Interlab G26 instrument (Interlab Sebia, Rome, Italy) and commercially available agarose plates for electrophoresis (Interlab Electrophoresis) according to the Manufacturer’s instructions.

*Serum concentrations of albumin, transferrin, alpha-2-macroglobulin and ceruloplasmin* were measured by the biuret method, using the COBAS c502 analyzer (Roche Diagnostics, Basel, Switzerland) and the dedicated reagents, calibrators and controls, at the Central Clinical Hospital Laboratory in Warsaw.

### Statistical analyses

Statistical analyses were performed using STATISTICA [StatSoft Inc. (2014) version 13]. The results are reported as mean ± SD, coefficient of variation (CV), median and range. Comparisons of the serum concentrations of thyroid hormones, proteins contained in the electrophoretic fractions and individual proteins between trimesters were made using the ANOVA with POST-HOC test (Tukey–Kramer). The Spearman`s test was used to calculate the coefficients of correlation. The value of p < 0.05 was considered to be statistically significant.

### EEthical approval

This study was approved by the Medical Ethics Committee at the Central Clinical Hospital of the Ministry of the Interior and Administration, Warsaw, in accordance with the Declaration of Helsinki, Decision No 71/2011.

### Consent to participate

Informed consent for participation in the study was obtained from each pregnant subject. Informed consent for participation in the study was obtained from parents and/or legal guardians of the subjects.

### Consent for publication

All authors have seen and approved the manuscript being submitted.

## Results

Variations in the concentrations of the thyroid hormones (TSH, fT3, fT4, fT3/fT4) and the concentrations of proteins contained in the electrophoretic fractions (albumin, alpha-1 globulins, alpha-2 globulins, beta-1 globulins, beta-2 globulins and gamma-globulins) measured in the sera of pregnant women in each trimester of pregnancy are presented in Table 1.

**Table 1:** Changes in the serum concentrations of thyroid hormones and six major protein fractions across normal pregnancy.

Parameter	Mean ± SD, (*CV*)*, median, range			p ANOVA
	Trimester of pregnancy			
	First (n = 55)	Second (n = 42)	Third (n = 39)	
TSH [µIU/dl]	**211 ± 205** (*97*%) 173 (10–1100)	**205 ± 157** (*76*%) 182 (7–1056)	**214 ± 88** (*48*%) 204 (33–415)	p = 0. 138
fT4 [ng/dl]	**1.19 ± 0.17** (*14*%) 1.21 (0.71–1.50)	**1.02 ± 0.12 (***13*%) 0.99 (0.77–1.27)	**0.90 ± 0.15** (*17*%) **0.88** (0.64–1.41)	I vs II p = 0.000 I vs III = 0.000 II vs III p = 0.008
fT3 [ng/dl]	**0.31 ± 0.04** (*11*%) 0.31 (0.22–0.39)	**0.28 ± 0.04** (*16*%) 0.28 (0.21 –0. 44)	**0.27 ± 0.03** (*12*%) 0.27 (0.22–0.35)	I vs II p = 0.002 II vs III p = 0.000
fT3/fT4	**0.26 ± 0.04** (*16*%) 0.26 (0.17–0.37)	**0.28 ± 0.05** (*18*%) 0.27 (0.19–0.40)	**0.31 ± 0.06** (*19.81*%) 0.32 (0.17–0.43)	I vs III p = 0.000 II vs III p = 0.039
Albumina [g/L]	**36.1 ± 3.4** (*9*%) 36.0 (27.5–42.3)	**31.2 ± 2.7** (9%) 31.0 (25.7–39.6)	**29.6 ± 2,4** (*8*%) 29.5 (24.6–35.4)	I vs II p = 0.000 I vs III p = 0.000
Alpha-1-globulin [g/L]	**2.3 ± 0.5** (20%) 2.3 (0.5–3.5)	**2.6 ± 0.4** (*14*%) 2.7 (1.6–3.3)	**2.7 ± 0.4** (*13*%) 2.7 (2.0–3.4)	I vs II p = 0.001 I vs III p = 0.000
Alpha-2-globulin [g/L]	**9.9 ± 1.3** (*13*%) 9.7 (7.4–13.3)	**10.5 ± 1.2** (*11*%) 10.6 (7.7–12.7)	**10.4 ± 1.3** (*12*%) 10.4 (7.0–12.4)	p = 0.056
Beta-1-globulin [g/L]	**6.6 ± 1.2** (*18*%) 6.5 (4.8–11.3)	**7.0 ± 1.0** (*14*%) 6.8 (5.1–9.5)	**7.4 ± 1.1** (*14*%) 7.6 (4.5–9.7)	I vs III p = 0.000
Beta-2-globulin [g/L]	**4.8 ± 0.9** (*18*%) 4.8 (3.2–7.2)	**4.5 ± 1.0** (*21*%) 4.4 (2.8–6.6)	**4.4 ± 0.8** (*18*%) 4.4 (2.1–6.1)	p = 0.141
Gamma-globulin [g/L]	**10.6 ± 2.4** (*22*%) 10.3 (5.9–18.9)	**8.2 ± 1.8** (*22*%) 7.5 (4.5–12.4)	**7.0 ± 2.1** (*30*%) 6.6 (3.2–11.7)	I vs II p = 0.000 I vs III p = 0.00

No statistically significant changes in the TSH concentrations were established between trimesters, but the high coefficients of variation (CV) were found to gradually decrease. The CV values indicate the highest variability of TSH concentration compared to the variability of other parameters of thyroid function. The dynamics of changes in the concentrations of fT4 and fT3 and the fT3/fT4 ratio differ between trimesters with a consistent decrease in the concentrations of fT4 across pregnancy, a decrease in the concentrations of fT3 from the first to second trimester of pregnancy and an increase in the fT3/fT4 ratio from the second to third trimester. The concentrations of proteins contained in particular electrophoretic fractions changed over time at different rates. There was a significant fall in the concentrations of albumin and gamma-globulins from the first to second trimester and a slow growth in the concentrations of proteins contained in the alpha-1-, alpha-2- and beta-1 globulin fractions with a significant increase in the concentrations of alpha-1-globulins from the first to second trimester and of beta1-globulins from the first to third trimester.

Table 2 presents the significant correlation coefficients (p < 0.05) between changing TSH, fT4 and fT4 concentrations, and fT3/fT4 ratios and the concentrations of constituent components of the electrophoretic protein fractions in maternal sera in the consecutive trimesters of pregnancy.

**Table 2:** Correlations between thyroid hormone levels and the serum concentrations of six major protein fractions across normal pregnancy.

Parameter	Trimester of pregnancy		
	First (n = 55)	Second (n = 42)	Third (n = 39)
**TSH**	p > 0.05	p > 0.05	**with alpha-2-globulin:** r = −0.41, p = 0.016 **with beta-1-globulin:** r = −0.34, p = 0.03
**fT4**	**with albumin:** r = 0.29, p = 0.024	**with albumin:** r = 0.52, p = 0.001	**with albumin:** r = 0.42, p = 0.010
**fT3**	p > 0.05	**with alpha-2-globulin:** r = −0.35, p = 0.018	p > 0.05
**fT3/fT4**	**with albumin:** r = −0.31 p = 0.016	**with albumin:** r = −0.32, p = 0.042 **with alpha-2-globulin:** r = −0.40, p = 0.009	**with albumin:** r = −0.44, p = 0.006

Significant relationships (p <0.05) are marked in bold.

Three electrophoretic protein fractions – albumin, alpha-2- and beta-1-globulins—were found to have significant associations with the thyroid panel. Throughout pregnancy there was a consistent positive association between albumin and fT4 and a negative association of albumin with the fT3/fT4 ratio. Additionally, in the second and third trimesters there were significant associations of alpha-2- and beta-1-globulins with TSH, fT3 and fT3/fT4 ratio.

Table 3 presents changing concentrations of individual proteins as representative for selected electrophoretic fractions – ALB (albumin fraction), CER and AMG (alpha-2-globulin fraction) and TRF (beta-1-globulin fraction). These representative proteins were chosen based on the literature data confirming their electrophoretic localization^11^.

**Table 3:** Changes in the serum concentrations of albumin (ALB), transferrin (TFR), ceruloplasmin (CER) and alpha-2-macroglobulin (AMG) across normal pregnancy.

Parameter	Serum protein concentrations mean ± SD, (*CV*)*, median, range			p ANOVA
	Trimesters of pregnancy			
	First (n = 55)	Second (n = 42)	Third (n = 39)	
ALB [g/dl]	**4.28 ± 0.29** (7%)	**3.72 ± 0.24** (6%)	**3.58 ± 0.18** (5%)	p < 0.0001
	4.25 (3.32–4.90)	3.70 (3.40–4.25)	3.58 (3.13–3.88)	
TRF [mg/dl]	**289.7 ± 54.1** (19%)	**327.8 ± 60.4** (18%)	**381.2 ± 55.5** (15%)	p < 0.0001
	282.5 (203.0–467.0)	326.0 (233.0–487.0)	380.0 (274.0–493.0)	
CER [mg/dl]	**32.90 ± 8.52** (26%)	**43.33 ± 5.91** (14%)	**44.08 ± 5.54** (13%)	p < 0.0001
	32.00 (17.00–57.00)	44.00 (29.00–57.00)	45.00 (33.00–57.00)	
AMG [mg/dl]	**211.1** ± 50.8 (24%)	**235.3 ± 45.2** (19%)	**209.6 ± 53.3** (25%)	p = 0.053
	199.0 (120.0–336.0)	233.0 (141.0–347.0)	205.0 (98.0–326.0)	

*CV—coefficient of variation.

ALB concentrations gradually decreased with gestation length unlike the concentrations of TRF and CER which increased while the concentrations of AMG were slightly fluctuating.

Table 4 shows correlations of the thyroid panel with the changing concentrations of ALB, CER, TFR and AMG.

**Table 4:** Correlations between thyroid hormone levels and the serum concentrations of ALB, TRF, CER and AMG across normal pregnancy.

Thyroid parameter	Coefficients of correlation with serum protein concentrations*		
	Trimesters of pregnancy		
	First (n = 55)	Second (n = 42)	Third (n = 39)
**TSH**	No correlation p > 0.05	No correlation p > 0.05	No correlation p > 0.05
**fT4**	***with ALB*:** r = 0.410, p = 0.004 ***with TRF*:** r = −0.310, p = 0.010	***with ALB***: r = 0.330, p = 0.025 ***with TRF*:** r = −0.297, p = 0.074	***with ALB*:** r = 0.380, p = 0.030 ***with TRF*:** r = −0.360, p = 0.040 ***with AMG*:** r = 0.450, p = 0.010
**fT3**	No correlation p > 0.05	***with AMG*** *r* = *−0.386, p* = *0.018* ***with CER*:** *r* = *−0.345, p* = *0.031*	No correlation p > 0.05
**fT3/fT4**	***with ALB*:** r = −0.310, p = 0.020	***with ALB*:** r = −0.303, p = 0.051 ***with AMG*:** r = −0.410, p = 0.034 ***with CER*:** r = −0.420, p = 0.008	***with ALB*:** r = −0.340, p = 0.036 ***with AMG*:** r = −0.426, p = 0.022 ***with TRF*:** r = 0.492, p = 0.006

Significant relationships (p <0.05) are marked in bold.

*Spearman test.

The correlations between TSH, fT4, fT3 and fT3/fT4 ratio and the concentrations of individual proteins differ between trimesters. No correlation was established between TSH and changing concentrations of the four proteins. In all trimesters of pregnancy, the concentrations of fT4 were correlated positively with ALB and negatively with TRF. The effect of fT3 and fT3/fT4 was observed in the second and third trimesters when it were negatively correlated with AMG and CER.

**Figure 1:**
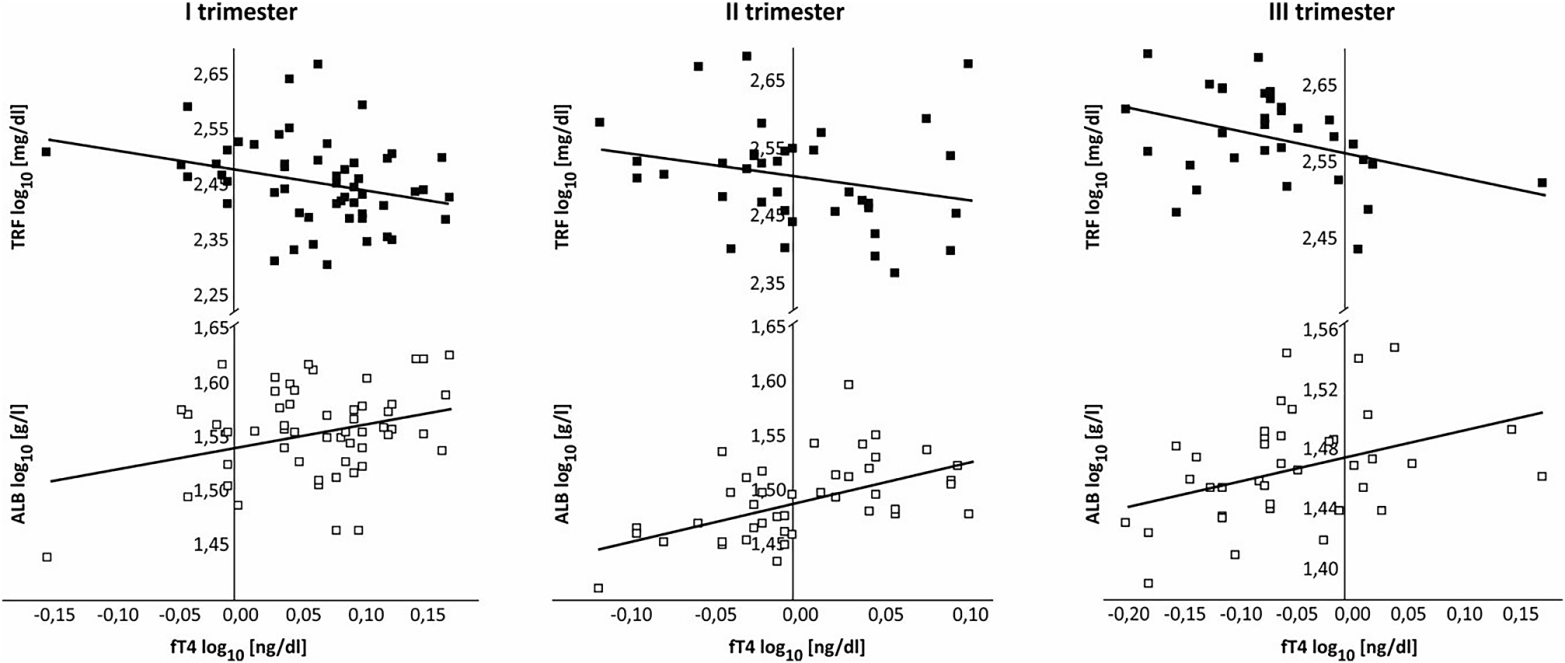
Correlations between concentrations of fT4 and of ALB (open square) and TRF (filled square) in maternal serum across pregnancy.

Figure 1 graphically represents the correlations between the serum concentrations of fT4 and of ALB and TFR in consecutive trimesters of pregnancy. Changes in the concentrations of ALB and of TRF regulated by changing fT4 levels were found to move in opposite directions throughout pregnancy.

## Discussion

The results demonstrate associations between the concentrations of serum proteins and the thyroid panel undergoing changes in consecutive trimesters of pregnancy. Significant associations were demonstrated between the concentrations of albumin, alpha-2- and beta-1-globulins separated by a preliminary serum protein electrophoresis and the levels of TSH, fT4, fT3, and the fT3/fT4 ratio. Four proteins (ALB, TRF, CER and AMG) were selected as representative for specific electrophoretic fractions and used in further immunoassays. These representative proteins were selected from 22 most abundant serum proteins, with well characterized electrophoretic localizations and biological properties described in the literature^3, 11–14^. Serum concentrations of ALB (albumin fraction), TRF (beta-1-globulin fraction), and CER and AMG (alpha-2-globulin fraction) demonstrated different rates of change from trimester to trimester which may reflect differences in their involvement in metabolic processes associated with consecutive stages of pregnancy.

The amount of TSH in the blood is a parameter of thyroid function routinely used to identify thyroid disorders^3^, but its association with the concentrations of serum proteins was weak, with the exception of the alpha-2- and beta-1-globulin fractions in the third trimester only.

No correlation was established throughout pregnancy between the levels of TSH and changing concentrations of ALB, TRF, CER and AMG.

Interestingly, the associations observed for the concentrations of fT4 vs ALB and fT4 vs TRF, maintained in the three trimesters of pregnancy, move in opposite directions. The finding we report of a positive association between fT4 and ALB serum concentrations may provide novel, clinically useful information about the underlying physiological mechanisms of some pregnancyassociated processes or the causes of abnormalities. ALB, along thyroxine-binding globulin (TBG) and transthyretin, is a major transport protein transporting thyroid hormones in the vascular bed. TBG concentration is negatively correlated with ALB concentration which means that a significant increase in the concentration of TBG in pregnancy leads to decreases in the concentrations of ALB^2, 15^. A slight decrease in the serum concentration of ALB in pregnancy is considered normal^16^, but significantly lower ALB levels in the third trimester of pregnancy were associated with increased maternal mortality and morbidity^11^. In pregnancy, serum albumin levels may be decreased in cases of renal insufficiency with proteinuria, preeclampsia, gestational hypertension and gestational edema. Therefore, it may be hypothesized that proteinuria and low serum ALB may lead to low fT4 levels with adverse effects on the course of pregnancy^11, 15, 17–21^.

A negative correlation of fT4 with TFR we observed is consistent with the finding that iron metabolism and thyroid functions are interdependent^22^. Iron deficiency has been linked to hypothyroidism and high TRF and vice versa, low TRF reflects excess iron stores in hyperthyroidism. TRF delivers iron to tissues, due to its iron-binding capacity is considered an important regulator of iron levels in the body, it maintains oxidant/anti-oxidant balance and has critical role in host defence by depriving iron from invading pathogens and as an immune regulator during acute inflammation^23, 24^.

The mechanism of the negative association of fT4 with TFR levels found in this study in sera from pregnant women remains unclear. Reports by other authors point to high iron stores as a risk factor of type 2 diabetes mellitus and gestational diabetes mellitus (GDM) which may develop as early as the first trimester. GDM is a very common metabolic disorder in pregnancy but the mechanism underlying a relationship between excess body iron and GDM has not been elucidated^25^. The authors of a population-based cohort study suggest that hypothyroidism may be associated with risk of type 2 diabetes while gradually increased fT4 levels decrease the risk^26^. The findings in the present study suggest assumptions that require additional explanations for the pathomechanism of the negative linkage of increased fT4 with decreased TRF. Such an observation may indicate their shared biological role producing a combined protective effect against GDM associated with excess body iron. A question arises whether the evaluation of fT4 vs TFR would provide additional arguments to solve an existing controversy^24^ over routine prophylactic iron supplementation in all pregnant women without earlier laboratory investigations to confirm its actual need.

The above observations suggest novel laboratory investigations to assess maternal health in pregnancy, using the association of serum fT4 concentrations with changes in iron levels and its metabolism, and ALB concentrations. According to the literature, in hypothyroidism, low fT4 may suggest the effect of iron depletion leading to the increased serum concentrations of TRF^26, 27^. Low fT4 levels have been also linked to low ALB, which is widely used to determine nutritional status^28–30^. The association of low ALB levels and high TRF levels with low fT4 levels in maternal serum we observed may serve as a diagnostic panel to confirm altered nutritional status in malnutrition.

A negative correlation was established in the second trimester of pregnancy between serum fT3 and alpha-2-globulins. The negative correlations of fT3 with CER and AMG, high abundance proteins located in this fraction may indicate their interaction in ongoing metabolic processes. As shown in the results we present, AMG concentrations changed only slightly across pregnancy, although the presence in pregnant women of pregnancy zone protein (PZP) which is part of the alpha-2-globulin fraction might have contributed to the observed increases. AMG, a 720 kDa tetramer and PZP, a 360 kDa dimer are strongly homologous glycoprotein proteinase inhibitors of human plasma^30^. AMG functions as a major endoprotease inhibitor and its concentrations are decreased in hypothyroidism^1, 7, 31^. CER is an intravascular antioxidant and can function as a free radical scavenger. Thyroid hormones are involved in both production and elimination of reactive oxygen species (ROS). In hyperthyroidism, oxidative stress increases proportionally to the degree of thyroid overactivity while in hypothyroidism decline in ROS generation is associated with antioxidant activity. GDM has been identified as an inductor of oxidative stress and ROS production^32, 33,34^.

The characteristic relationships between the variability of the concentration of thyroid hormones and individual serum proteins including those binding thyroid hormones raise important questions for clinicians about the casual relationships between these parameters during pregnancy. Understanding the mechanisms of the interrelationship between the concentrations of thyroid hormones and estrogen-dependent serum proteins can provide knowledge about their joint participation in the regulation of metabolic processes typical of the course of pregnancy and facilitate the distinction between physiological and pathological states. during this period.

The limitation of the study was the differences in the number of samples obtained from the examined women in the subsequent stages of pregnancy. From 15 of 65 participants 3 serum samples were obtained representing each trimester.

In conclusion, changes in the thyroid panel observed during pregnancy demonstrate the association of thyroid hormones with the concentrations of proteins in the albumin and alpha-2- and beta-1-globulin fractions. ALB, TRF, CER and AMG are individual proteins in these fractions and are specifically associated with changes in serum levels in thyroid hormones in pregnancy. Published studies emphasize potential effects of even mild subclinical thyroid dysfunction on detrimental pregnancy outcomes. A novel panel of serum proteins specifically associated with thyroid hormones may be an alternative method to diagnose changes in thyroid function in pregnancy and a potential source of novel biomarkers to identify the effects on maternal metabolism during pregnancy.

## Abbreviations

ALB: Albumin
TRF: Transferrin
AMG: Alpha-2-macroglobulin
CER: Ceruloplasmin
TBG: Thyroxine binding globulin
GDM: Gestational diabetes mellitus
ROS: Reactive oxygen species
PZP: Pregnancy zone protein
fT3: Free tri-iodothyronine
fT4: Free thyroxine
